# Analysis of cardiovascular mortality, bleeding, vascular and cerebrovascular events in patients with atrial fibrillation vs. sinus rhythm undergoing transfemoral Transcatheter Aortic Valve Implantation (TAVR)

**DOI:** 10.1186/s12872-017-0736-6

**Published:** 2017-12-20

**Authors:** Joerg Herold, Vasiliki Herold-Vlanti, Mohammad Sherif, Blerim Luani, Christin Breyer, Klaus Bonaventura, Ruediger Braun-Dullaeus

**Affiliations:** 1 Department of Internal Medicine/Cardiology and Angiology, Otto-von-Guericke University of Magdeburg, Leipziger Str. 44, 39120 Magdeburg, Germany; 2 Department of Internal Medicine/Cardiology and Angiology, University of Rostock, Ernst-Heydemann-Straße 6, 18057 Rostock, Germany; 3 Department of Internal Medicine/Cardiology and Angiology, Ernst-von-Bergmannstrost Clinic, Charlottenstraße 72, 14467 Potsdam, Germany

**Keywords:** TAVR, VARC-2, Sinus rhythm, NOAC, Bleeding, Atrial fibrillation, Antiplatelet therapy

## Abstract

**Background:**

Transcatheter aortic valve replacement (TAVR) has been demonstrated to be an established therapy for high-risk, inoperable patients with severe symptomatic aortic valve stenosis. For patients with moderate surgical risk, TAVR is equivalent to conventional aortic valve surgery. However, atrial fibrillation (AF) is also present in many of these patients, thus requiring post-implantation oral anticoagulation therapy in addition to the inhibition of thrombocyte aggregation, which poses the risk of bleeding complications. The aim of our work was to investigate the influence of AF on mortality and the occurrence of bleeding, vascular and cerebrovascular complications related to TAVR according to the VARC-2 criteria.

**Methods:**

Two hundred eighty-three patients who underwent TAVR between March 2010 and April 2016 were retrospectively examined. In total, 257 patients who underwent transfemoral access were included in this study. The mean patient age was 81 ± 6 years, 54.1% of the patients were women, and 42.4% had pre-interventional AF.

**Results:**

Compared to patients with sinus rhythm (SR, *n* = 148), patients with AF (*n* = 109) had an almost three-fold higher incidence of major vascular complications (AF 14.7% vs. SR 5.4%, *p* = 0.016) and life-threatening bleeding (AF 11.9% vs. SR 4.1%, *p* = 0.028) during the first 30 post-procedural days. However, the rate of cerebrovascular complications (AF 3.7% vs. SR 2.7%, *p* = 0.726) did not significantly differ between the two groups. Overall mortality was significantly higher in patients with AF during the first month (AF 8.3% vs. SR 2.0%, *p* = 0.032) and the first year (AF 28.4% vs. SR 15.3%; *p* = 0.020) following TAVR.

**Conclusion:**

Patients with AF had significantly more severe bleeding complications after TAVR, which were significantly related to mortality. Future prospective randomized studies must clarify the optimal anticoagulation therapy for patients with AF after TAVR.

**Trial registration:**

DRKS00011798 on DRKS (Date 17.03.2017).

## Background

Degenerative aortic stenosis is the most common form of heart valve disease affecting the elderly population [1]. Transcatheter aortic valve implantation (TAVR) has been shown to be superior to medical therapy for inoperable patients and is not inferior to surgical aortic valve replacement (SAVR) in terms of all-cause mortality [2–5]. Five-year data from the PARTNER study recently confirmed the long-term success of the TAVR strategy [4, 6, 7].

However, patients undergoing TAVR are also at high risk for both bleeding and stroke complications. The mechanisms of peri-procedural bleeding complications appear to be mainly related to vascular/access site complications, whereas the pathophysiology of cerebrovascular events remains largely unknown. Life-threatening bleeding complications may also dramatically increase mortality [8, 9].

Nevertheless, among elderly patients for whom TAVR is indicated, prescreening often reveals other comorbidities. Both aortic valve stenosis and atrial fibrillation (AF) are particularly common among patients in the 8th decade of life [10, 11]. Managing these patients, who require oral anticoagulation to address AF-associated increased thromboembolic risk and who undergo TAVR due to severe aortic valve stenosis, presents a challenge in clinical practice. In particular, the question arises regarding whether to administer anticoagulant therapy after TAVR to prevent vascular complications or bleeding and cerebrovascular events. No consistent evidence-based anticoagulant therapy recommendations are currently available for patients with AF after TAVR. The aim of this retrospective study was to examine the influence of AF in a “real world” TAVR cohort.

## Methods

### Study design and patients

**Fig. 1:**
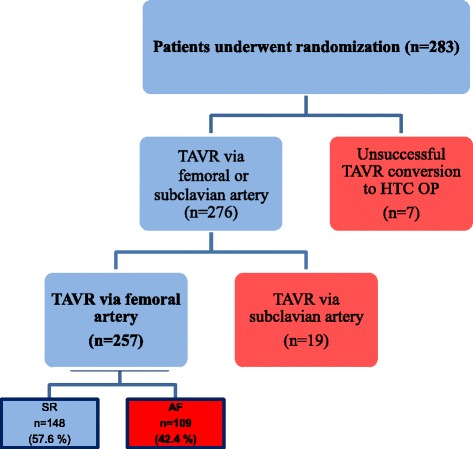
Trial enrollment showing the total population of patients who received a TAVR procedure from 03/2010 to 04/2016. Patients without a successfully implanted TAVR (*n* = 4, 1.4%) or patients who had to be converted to open cardiac surgery (HTC-OP; *n* = 3; 1.1%) were excluded. Due to the small group size, the patients who received a TAVR over the subclavian artery (*n* = 19; 6.7%) were not included in the study. Thus, 257 patients with transfemoral access were included in the further analysis. Visualization of the classification of the patient collective according to the preprocedural cardiac rhythm: Almost half of the total patient population (42.4%) suffered from AF

A total of 283 patients underwent TAVR between March 2010 and April 2016 at the University Hospital of Cardiology and Angiology of Otto-von-Guericke-University Magdeburg. The trial was registered at German Clinical Trials Register (DRKS) (DRKS00011798). The access route to the aortic valve was through the femoral artery and the subclavian artery. Patients whose implantation procedures were discontinued (frustrated TAVR, *n* = 4; 1.4%) and those requiring immediate cardiac surgery due to acute life-threatening intraprocedural complications (*n* = 3; 1.1%) were excluded from further analysis. Patients with transsubclavian TAVR (*n* = 19; 6.7%) were not included in the evaluation because this subgroup was too small for statistical calculations (Fig. 1). Thus, 257 patients were retrospectively divided into SR and AF groups according to pre-procedural cardiac rhythm. The presence of AF prior to TAVR was determined electrocardiographically and/or on the basis of outpatient or inpatient records. The two groups were compared for baseline characteristics, post-procedural mortality and complications, and vascular and cerebrovascular complications within 30 days, 3 months, 6 months, and 1 year after TAVR. The complications following TAVR were documented using the VARC-2 criteria [12]. To evaluate the use of coagulation inhibitors in patients with AF after TAVR, patients with AF were divided post-procedurally into subgroups. Because most patients with AF were treated with either vitamin K antagonist (VKA) plus clopidogrel or with a NOAC plus clopidogrel for 3 months, these two subgroups were compared for mortality and complication rates.

### Medication

The patients with AF and TAVR received triple therapy (phenprocoumon/clopidogrel (75 mg o.d./aspirin 100 mg o.d.)), phenprocoumon/clopidogrel (75 mg o.d.) or a combination of a NOAC and clopidogrel (75 mg o.d.) for 3 months. Bleeding complications were defined by the VARC- 2 criteria.

### Endpoints

Clinical follow-up, including echocardiography, electrocardiography, and laboratory testing for creatinine, transaminases, red blood cells and thrombocytes was performed before and after valve implantation prior to discharge and at 1, 3, 6, 9 and 12 months following the procedure.

### Statistical analyses

The statistical data analyses were performed using SPSS 24.0 (SPSS Inc., Chicago, USA). The results were presented both descriptively and graphically. Continuous values were presented as mean values with standard deviations and are represented by box plots for the individual groups being examined. Categorical variables were presented as absolute and relative frequencies of the overall study population and by using bar graphs. A comparison of the groups (AF vs. SR) for statistically significant differences was performed using the unpaired t-test (Satterthwaite) for continuous variables and Fisher’s exact test for categorical variables. In the case of strong deviations from a normal distribution, a natural logarithm transformation was used to facilitate use of the t-test. Survival curves were calculated using the Kaplan-Meier method, and the groups were compared using the log-rank test, taking censored data into account. Additionally, cumulative mortality was analyzed in the same manner as other complications without regard for censored data. Using the multivariate COX regression model, different baseline characteristics were examined for their prognostic significance in relation to survival probability and one-year overall mortality. In this process, influential variables were selected using stepwise conditional forward selection. The results were presented in the form of odds or hazard ratios, 95% confidence intervals and/or *p* values. The p values were calculated as exact two-tailed values. The significance level was set to 0.05, such that p values ≤ 0.05 indicated statistically significant results. All analyses were deliberately performed to the full level of significance. No corrections were performed for multiple comparisons due to the exploratory nature of this investigation.

## Results

### Patient characteristics

Overall, 283 patients were screened. According to the inclusion and exclusion criteria, 257 patients were eventually included. Nineteen screened patients were excluded because of subclavian access. TAVR was not successful in 7 of the 257 patients (2.4%). After these exclusions, 148 patients with SR vs. One hundred and nine with AF who underwent TAVR via transfemoral access were included for further analysis (Fig. 1).

The average age of the entire study group was 80.8 ± 6.0 years on the day of implantation, with 45.9% male patients (*n* = 118). The baseline characteristics of the entire patient group and the two groups classified according to preprocedural cardiac rhythm are summarized in Table 1. With regard to comorbidities, significantly more patients in the AF group than in the SR group had PAD (21.1% vs. 11.5%, respectively, *p* = 0.038). Furthermore, significantly more patients in the AF group had already received a pacemaker (SR 7.4% vs. AF 19.3%, *p* = 0.007). The remaining features were not significantly different between the two groups.

**Table 1:** Characteristics of the patients at baseline

Characteristics of the Patients at Baseline	Total population (*n* = 257)	SR-group (n = 148)	AF-group (n = 109)	*p* Value
Age — yr	80.8 ± 6.0	80.6 ± 6.0	80.9 ± 6.0	0.736
Male sex — no. (%)	118 (45.9%)	62 (41.9%)	56 (51.4%)	0.163
Body-mass index (kg/m^2^)	28.2 ± 5.5	28.3 ± 5.6	28.1 ± 5.3	0.724
Diabetes mellitus (n. %)	110 (42.8%)	57 (38.5%)	53 (48.6%)	0.126
Diabetes treated with insulin	68 (26.5%)	33 (22.3%)	35 (32.1%)	0.087
Hypertension	223 (86.8%)	126 (85.1%)	97 (89.0%)	0.457
Dyslipidemia (n. %)	179 (69.6%)	102 (68.9%)	77 (70.6%)	0.785
PAD	40 (15.6%)	17 (11.5%)	23 (21.1%)	0.038
Chronic obstructive pulmonary disease (n %)	50 (19.5%)	33 (22.3%)	17 (15.6%)	0.204
Medical history — no. (%)				
Chronic kidney disease on dialysis	8 (3.1%)	7 (4.7%)	1 (0.9%)	0.143
History of cancer	64 (24.9%)	40 (27.0%)	24 (22.0%)	0.384
Active cancer	20 (7.8%)	10 (6.8%)	10 (9.2%)	0.489
Coronary heart disease	210 (81.7%)	120 (81.1%)	90 (82.6%)	0.871
Coronary-artery bypass surgery	36 (14.0%)	18 (12.2%)	18 (16.5%)	0.365
Myocardial infarction	30 (11.7%)	15 (10.1%)	15 (13.8%)	0.433
PCI	67 (26.1%)	42 (28.4%)	25 (22.9%)	0.389
Pacemaker	32 (12.5%)	11 (7.4%)	21 (19.3%)	0.007
Stroke	40 (15.6%)	22 (14.9%)	18 (16.5%)	0.731
NYHA III	164 (63.8%)	96 (64.9%)	68 (62.4%)	0.695
NYHA IV	61 (23.7%)	31 (20.9%)	30 (27.5%)	0.238

All values correspond to the mean ± standard deviation or the number n (proportion in %). PAD was defined as claudication intermittens or any symptom corresponding to ≥ Fontaine stage II and / or as amputation in the context of an arterial occlusion and/or present or planned endovascular intervention for the revascularization of the peripheral vessels

### Laboratory parameters, echocardiographic characteristics and risk scores

**Fig. 2:**
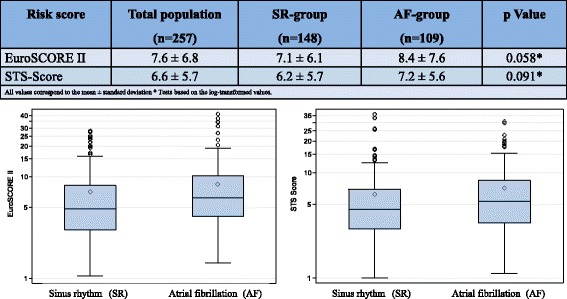
The risk assessment at baseline demonstrated patients with AF did not significantly differ from those with SR

Table 2 lists the laboratory parameters of the samples collected 2 days before valve implantation and the echocardiographic characteristics. These are listed as the mean values for the entire patient population and for the two groups in direct comparison. There were no statistically significant differences between the two groups. Concerning the baseline risk assessment, patients with AF did not significantly differ from those with SR (Fig. 2).

**Table 2:** Summary of the main parameters of the baseline laboratory and echocardiographic findings. There was no significant difference between the two groups regarding the laboratory and echocardiographic parameters

Baseline laboratory	Total population (n = 257)	SR-group(n = 148)	AF-group(n = 109)	*p* Value
Hemoglobin (mmol/l)	7.5 ± 1.1	7.5 ± 1.1	7.4 ± 1.1	0.227
Creatinin (umol/l^1^)	108.1 ± 44.1	103.5 ± 37.1	114.2 ± 51.3	0.121*
Glomerular filtration rate CKD-EPI (ml/min^1^)	54.5 ± 19.2	54.9 ± 17.9	54.0 ± 20.9	0.718
Thrombocyte (Gpt/l)	222.5 ± 75.6	225.3 ± 77.5	218.6 ± 73.2	0.479
Echocardiographic parameters	Total population (n = 257)	SR-group (n = 148)	AF-group (n = 109)	*p* Value
Aortic valve area (cm2)	0.72 ± 0.17	0.72 ± 0.17	0.71 ± 0.16	0.799
Pulmonary hypertension (PaSP >55 mmHg)	23 (8.9%)	11 (7.4%)	12 (11.0%)	0.379
Ejection fraction (%)	46.1 ± 13.8	47.2 ± 13.7	44.5 ± 13.9	0.113
Ejection fraction <40%	59 (23.0%)	30 (20.3%)	29 (26.6%)	0.293

^1^Patients not requiring dialysis (*n* = 8; 3.1% or SR: *n* = 7; 4.7% AF: *n* = 1, 0.9%). * Test using log-transformed values

All values correspond to the mean ± standard deviation or the number n (proportion in %)

### DAPT vs. triple therapy

Patients with AF received postoperative therapeutic heparinization (AF 51.4% vs. SR 4.1%, *p* < 0.001) and combination therapy consisting of either OAC and APT (AF 67.9% vs. SR 6.1%, *p* < 0.001) or triple therapy (AF 9.2% vs. SR 0.7%, *p* = 0.001). Conversely, DAPT was administered significantly more frequently to patients with SR (SR 93.2% vs. AF 16.5%, *p* < 0.001).

### Vascular complications

The incidence of major vascular complications was 9.3% overall during the first 30 days and differed significantly between the two groups. Specifically, almost three times as many major vascular complications occurred in patients with AF than in patients with SR (AF 14.7% vs. SR 5.4%, *p* = 0.016) (Table 3).

**Table 3:** Major vascular complications

Follow-up	Total population (*n* = 257)	SR-group(*n* = 148)	AF-group(n = 109)	*p* Value	OR (95% CI)
30 days	9.3% (24/257)	5.4% (8/148)	14.7% (16/109)	0.016	3.01 (1.24–7.32)
3 months	9.3% (24/257)	5.4% (8/148)	14.7% (16/109)	0.016	3.01 (1.24–7.32)
6 months	9.9% (24/241)	5.8% (8/137)	15.4% (16/104)	0.016	2.98 (1.22–7.26)
12 months	10.8% (24/219)	6.3% (8/124)	16.7% (16/95)	0.017	2.95 (1.20–7.22)

The table shows the major vascular complications of the total patient group and of the two groups over the course of the follow-up period. The parentheses list the number of major vascular complications and the number of patients available for analysis

In patients with AF, almost three times as many major vascular complications occurred during the first 30-day post-procedural complications than in patients with SR. No further increase in major vascular complications after the first 30 days were noted, as these complications mainly occurred around the intervention

Regarding minor vascular complications, a similar, non-significant difference was found between the two groups (Table 4). The temporal distribution of minor vascular complications was also correlated with the occurrence of major vascular complications (Table 4).

**Table 4:** The upper part (lite blue) summarizes the minor vascular complications of the total population and of the two groups. The parentheses list the number of minor vascular complications and the number of patients available for analysis. No significant difference was observed between the two groups with respect to minor vascular complications

	Total population (n = 257)	SR-group(n = 148)	AF-group (n = 109)	*p* Value	OR (95% CI)
Follow-up Minor vascular complication					
30 days	22.6% (58/257)	22.3% (33/148)	22.9% (25/109)	1.00	1.09 (0.61–1.96)
3 months	23.0% (59/257)	22.3% (33/148)	23.9% (26/109)	0.767	1.09 (0.61–1.96)
6 months	24.8% (60/241)	23.9% (33/137)	26.0% (27/104)	0.764	1.12 (0.62–2.01)
12 months	27.3% (60/219)	26.4% (33/124)	28.4% (27/95)	0.762	1.11 (0.61–2.01)
Follow-up Major bleeding					
30 days	3.5% (9/257)	4.1% (6/148)	2.8% (3/109)	0.737	0.67 (0.16–2.74)
3 months	3.9% (10/257)	4.7% (7/148)	2.8% (3/109)	0.525	0.57 (0.14–2.26)
6 months	4.5% (11/241)	5.8% (8/137)	2.9% (3/104)	0.360	0.48 (1.13–1.87)
12 months	5.5% (12/219)	7.2% (9/124)	3.2% (3/95)	0.240	0.42 (0.11–1.60)
Follow-up Minor bleeding					
30 days	19.8% (51/257)	21.6% (32/148)	17.4% (19/109)	1.00	1.09 (0.61–1.96)
3 months	19.8% (51/257)	21.6% (32/148)	17.4% (19/109)	0.767	1.09 (0.61–1.96)
6 months	21.0% (51/241)	23.0% (32/137)	18.3% (19/104)	0.764	1.12 (0.62–2.01)
12 months	23.1% (51/219)	25.4% (32/124)	20.0% (19/95)	0.762	1.11 (0.61–2.01)

The middle part of the table (blue) shows the major bleeding events of the total collective and of the two groups during the course of the follow-up period. The lower part of the table (deep blue) lists the minor bleeding events. The parentheses list the number of bleeding events and the number of patients available for analysis. With regard to the occurrences of major and minor bleeding, no significant accumulation was found in one of the two groups

### Major and minor bleeding

With regard to the occurrence of major and minor bleeding, no significant differences between the two groups could be demonstrated. However, patients with SR had more severe major and minor bleeding (Table 4).

### Life-threating bleeding

During the first 30 days, a total of 19 life-threatening secondary bleeding events were recorded in the overall patient group (7.4%). Patients with pre-interventional AF had nearly three times as many events as patients with SR (AF 11.9% vs. SR 4.1%, *p* = 0.028). This result corresponds to the rates and the temporal development of major vascular complications. Thus, most life-threatening bleeding occurred around the time of implantation and the changeover to OAC (NOAC or VKA) and DAPT. Once the patients became accustomed to the medication, there were no further significant changes (increases or decreases in bleeding) between the two groups (Table 5).

**Table 5:** Life-threatening bleeding

Follow-up	Total population (n = 257)	SR-group(n = 148)	AF-group(n = 109)	*p* Value	OR (95% CI)
30 days	7.4% (19/257)	4.1% (6/148)	11.9% (13/109)	0.028	3.21 (1.18–8.72)
3 months	7.8% (20/257)	4.7% (7/148)	11.9% (13/109)	0.057	2.73 (1.05–7.09)
6 months	8.2% (20/241)	5.0% (7/137)	12.5% (13/104)	0.057	2.69 (1.04–7.01)
12 months	9.0% (20/219)	5.6% (7/124)	13.5% (13/95)	0.057	2.66 (1.02–6.96)

Shows the life-threatening bleeding events of all patients and of the two groups during the course of the follow-up period. The parentheses list the number of life-threatening bleeding events and the number of patients available for analysis

Patients with AF showed nearly three times more life-threatening bleeding events than patients with SR. This result corresponds to the rates and the temporal development of major vascular complications. Most of the life-threatening bleeding occurred peri-precidually and during initiation of oral anticoagulation or dual anti-platelet therapy. Thereafter, no significant differences were determined

### Transfusion of erythrocyte concentrates

**Fig. 3:**
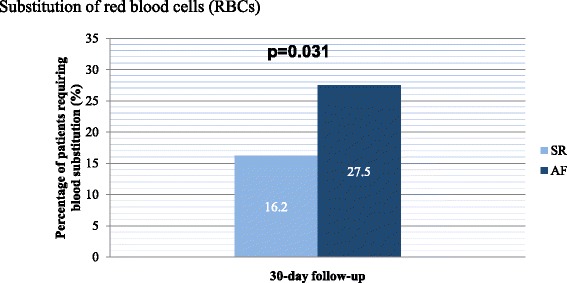
Illustrates the substitution of red blood cells (RBCs). Within the first 30 days, a blood transfusion with RBCs was required in approximately one of every five (21.0%) patients. Significantly more RBCs had to be administered to patients with AF

During the first 30 days, a blood transfusion with at least one erythrocyte concentrate (RBC) unit was required for approximately one of every five (21.0%) patients. The need for RBC transfusion was significantly different between the two groups, as significantly more patients with AF required RBC administration (AF 27.5% vs. SR 16.2%, *p* = 0.031, OR 1.96, 95% CI 1.07–3.60). The difference in RBC requirements between the two groups is shown schematically in Fig. 3.

### Stroke

The incidence of stroke within the first 30 days was 3.1% in the overall patient group. There was no significant difference between the two groups in stroke incidence during the first 30 days after TAVR (SR 2.7% vs. AF 3.7%, *p* = 0.726) or during the complete follow-up period (Table 6). There was also no significant difference in the incidence of transient ischemic attacks (TIAs) between the two groups (SR 0.0% vs. AF 1.8%, *p* = 0.179) within the first 30 days after TAVR.

**Table 6:** Cerebrovascular complications

Follow-upStroke	Total population (n = 257)	SR-group(n = 148)	AF-group(n = 109)	*p* Value	OR (95% CI)
30 days	3.1% (8/257)	2.7% (4/148)	3.7% (4/109)	0.726	1.37 (0.34–5.61)
3 months	3.1% (8/257)	2.7% (4/148)	3.7% (4/109)	0.726	1.37 (0.34–5.61)
6 months	3.3% (8/241)	2.9% (4/137)	3.8% (4/104)	0.728	1.34 (0.33–5.49)
12 months	5.0% (11*/219)	4.0% (5/124)	6.3% (6/95)	0.537	1.62 (0.48–5.47)

Lists the cerebrovascular complications up to the one-year follow-up. The strokes are shown for the entire patient population and for the two groups during the follow-up period. The parentheses list the number of strokes and the number of patients available for analysis. * Ten ischemic strokes and one hemorrhagic stroke. The occurrence of stroke in the two groups showed no significant difference

### Overall mortality and cardiovascular mortality associated with TAVR

**Fig. 4:**
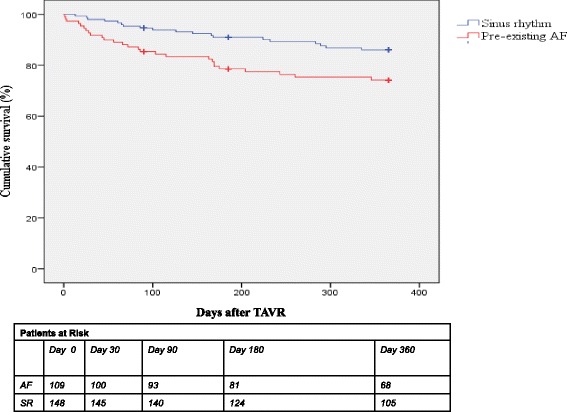
Kaplan-Meier curves of the one-year cumulative survival of patients with and without pre-existing atrial fibrillation (AF). Event rates were also calculated with the use of the log-rank test. Deaths from unknown causes were assumed to be deaths from cardiovascular cause. AF caused a higher overall mortality according to TAVR

**Fig. 5:**
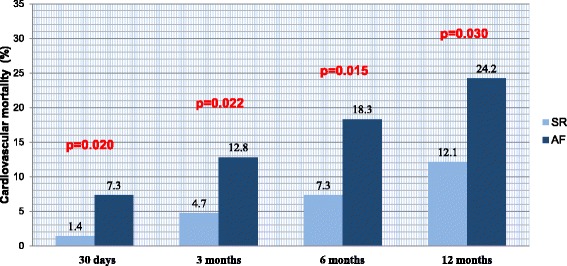
Shows the cardiovascular mortality rates up to the one-year follow-up. Patients with pre-existing AF presented a higher cardiovascular mortality after 30 days. This difference persisted for 12 months

The total patient population showed cumulative 30-day, 3-month, 6-month and one-year overall mortality rates of 4.7, 9.3, 14.9 and 21.0%, respectively. With respect to pre-interventional heart rhythms, patients with pre-existing AF had markedly higher cumulative mortality than patients in the SR group at 30 days (AF 8.3% vs. SR 2.0%, *p* = 0.032), 3 months (AF 14.7% vs. SR 5.4%, *p* = 0.016), 6 months (AF 22.1% vs. SR 9.5%, *p* = 0.010) and 1 year (AF 28.4% vs. SR 15.3%, *p* = 0.020). A statistically significant (*p* = 0.012) difference was found in AF patient mortality over the entire first year according to the Kaplan-Meier survival analysis (Fig. 4). Concerning cardiovascular mortality, the total patient population had cumulative 30-day, 3-month, 6-month and one-year cardiovascular mortality rates of 3.9, 8.2, 12.0 and 17.4%, respectively. Patients with pre-existing AF clearly had consistently higher cardiovascular mortality than patients in the SR group at 30 days (AF 7.3% vs. SR 1.4%, p = 0.020), 3 months (AF 12.8% vs SR 4.7%, *p* = 0.022), 6 months (AF 18.3% vs. SR 7.3%, *p* = 0.015) and 1 year (AF 24.2% vs. SR 12.1%, *p* = 0.030) (Fig. 5).

### Days of hospitalization

**Fig. 6:**
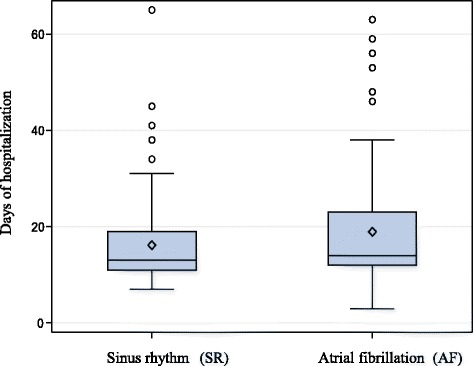
Patients with AF spent significantly more days in the hospital than patients with SR (SR 16.2 ± 9.2 vs. AF 18.9 ± 11.3 *p* = 0.043). * Test using log-transformed values

The average duration of hospitalization was 17.3 ± 10.2 days overall. Patients with AF had a significantly longer hospital stay than patients with SR (SR 16.2 ± 9.2 vs. AF 18.9 ± 11.3; *p* = 0.043) (Fig. 6).

### Predictors of one-year overall mortality after TAVR

Multivariate analysis revealed that pre-existing AF was an independent predictor of increased mortality at 1 year (HR: 2.10). All significant predictors for 1-year mortality are reported in Table 7.

**Table 7:** Shows the significant independent predictors for mortality by the 1-year follow-up according to the multivariate logistic regression

Significant predictors	Multivariate data analysis at baseline	
	Hazard Ratio (95% Confidence Interval)	*p* Value
Pre-existing AF	2.10 (1.17–3.78)	0.013
Anemia (Hb < 10.5 g/dL)	2.39 (1.28–4.47)	0.007
STS-score	1.08 (1.04–1.12)	< 0.001
Previous coronary artery bypass graft surgery (CABG)	0.25 (0.08–0.83)	0.024
Previous coronary artery disease (CAD)	3.27 (1.00–10.72)	0.050

## Discussion

Following the first aortic value implantation in 2002, TAVR was characterized by Cribier et al. [13] as an established therapy for high-grade, symptomatic aortic valve stenosis [14]. Furthermore, recently published data from the PARTNER-2 and SURTAVI studies show that TAVR is equivalent to conventional aortic valve replacement surgery for patients who present with intermediate surgical risk [7, 15]. In addition to other comorbidities, pre-existing AF is present in a significant proportion of elderly patients with high-grade degenerative aortic valve stenosis, with a rapidly increasing prevalence with increasing age [16, 17]. Because of the multi-morbidity of patients who often exhibit elevated bleeding risk, medical treatment after TAVR remains challenging. In practice, the limited dual platelet inhibition with aspirin and clopidogrel as an antithrombotic therapy has been most widely accepted according to TAVR and SR. The European Society of Cardiology (ESC) recommendations for IIa C indicate that dual antiplatelet therapy should be considered for the first 3–6 months after TAVR, followed by lifelong single antiplatelet therapy in patients who do not need oral anticoagulation for other reasons, whereas single antiplatelet therapy may be considered after TAVR in patients with high bleeding risk (III B) [18]. Nevertheless, there are clinical challenges in the management of patients with AF and TAVR. According the ESC guidelines, triple therapy for longer than 1 month should be considered for patients with a high risk of ischemia, which outweighs bleeding risk (IIa B); on the contrary, dual therapy comprising VKA and clopidogrel should be considered as an alternative to 1-month triple antithrombotic therapy (IIa A) in patients with high bleeding risk [18].

However, there are still uncertainties in both the clinical implementation and in the duration of triple-therapy or use of NOAC for patients with AF. The combination of anticoagulation with an antiplatelet therapy is used to prevent stroke and valve thrombosis but likely increases bleeding complications.

Therefore, 257 patients were included in this study between March 2010 and April 2016. Regarding pre-procedural cardiac rhythm, nearly half of the patients (*n* = 109; 42.4%) had AF. Therefore, the question arose as to whether the patients were randomly distributed into the SR and AF groups in this study. As previously mentioned, AF is the most frequent cardiac arrhythmia. Based on its age-dependent prevalence, the average age of those affected is between 75 and 85 years [16]. Additionally, the occurrence of AF is likely due to a high number of pre-valvular cardiac diseases, such as arterial hypertension, coronary heart disease and cardiac valve disease [17]. The increased incidence of AF in this patient population is therefore predictable given the mean study population age of 80.8 ± 6.0 years, comorbidity rates of 81.7% for coronary heart disease and 86.8% for arterial hypertension, and above all, the fact that all patients had high-grade aortic valve stenosis.

Although this was a retrospective survey rather than a prospective study, the baseline characteristics differed in only two parameters. Specifically, previous pacemaker implantation was significantly more common among patients with AF. In one sense, this might confer a prophylactic effect against AF in these patients. For the entire patient population, the EuroSCORE II was 7.6 ± 6.8 and the STS score was 6.6 ± 5.7, similar to the scores in the largest German registry (German Aortic Valve Registry, GARY). Current data from the GARY registry also show that more patients with low-risk scores underwent TAVR in recent years (the mean STS score according to the GARY registry was 5.0) [19].

Vascular complications during transfemoral TAVR cover a wide range of vascular injuries, such as perforations, ruptures, dissections and their sequelae involving access vessels or the aorta [20, 21]. Vascular complications were subdivided into major and minor complications according to VARC-2 criteria [22] and were compared between the AF and SR groups. The overall incidence of major vascular complications during the first 30 postoperative days was 9.3%. However, it is important to note that no further increase in vascular complications was observed after the first 30 days, as these events were mainly peri-procedural.

Due to the different recruitment periods of the patients included in the study, the use of different valve generations and valve implantation systems, and the different definitions of vascular complications depending on the study situation, existing published data on vascular complications differ considerably among large TAVR studies and registries. For example, in the PARTNER study, the rate of major vascular complications during the first 30 post-interventional days was as high as 16.2% using the modified VARC criteria [3, 5]. In the PARTNER-2 study, the rate of vascular complications according to the advanced VARC-2 criteria decreased to only 7.9% and to 8.5% for the transsubclavian and transfemoral cohorts, respectively [15]. The FRANCE-2 trial and SOURCE-XT registries showed major vascular complication rates (according to VARC-2 criteria) of 5.5% and 7.9%, respectively, for transfemoral access [23, 24]. A major vascular complication rate (according to VARC criteria) of 10.9% was reported in the ADVANCE study, predominantly with transfemoral implanted valve prostheses [25]. In the GARY registry, however, a low incidence of major vascular complications (4.1%) was observed. This can be explained by the fact that in the GARY registry, as in other studies, vascular complications are classified using different classification criteria than the VARC criteria. The GARY registry detects and divides complications into three categories. For example, aortic dissection and annular rupture after TAVR are classified as severe vital complications rather than as vascular complications [19]. Furthermore, the VARC-2 criteria significantly differ from the VARC-1 criteria in the definition of major vascular complications. These differences must be accounted for when interpreting the study results. For example, when using the VARC-2 criteria, any vascular injury that leads not only to life-threatening bleeding but also to major hemorrhage is recorded as a major vascular complication. Additionally, any unplanned endovascular or surgical intervention associated with a severe event such as death, life-threatening or major hemorrhage, visceral ischemia, or neurological impairment is defined as a major vascular complication according to the VARC-2 criteria [22, 26]. In an analysis of 403 consecutive TAVR patients, Steinvil et al. 0reported that use of the VARC-2 criteria led to a higher reported rate of major vascular complications than use of the VARC-1 criteria due to the detection of major bleeding [8]. Therefore, directly comparing the major vascular complication rate in our study population with the rates from previous large studies is difficult.

This study clearly shows that patients with AF have nearly three times more major vascular complications within the first 30 days after TAVR than patients with SR. Therefore, special attention should be paid to patients with AF during pre-procedural consultation and during the peri-procedural period in consideration of the significantly higher rates of major vascular complications and life-threatening bleeding.

Another relevant point is that there were more patients with PAD in the AF group. The association of PAD with significantly more frequent major vascular complications may be associated with the poorer vascular access in this patient group. Sinning et al. [27] observed that patients with PAD had a two-fold higher rate of major vascular complications after TAVR compared to patients without PAD.

Because most peri-procedural vascular injuries lead to bleeding, it can be assumed that these events are more pronounced in patients with AF than in patients with SR. To date, few studies have investigated vascular complications or bleeding after TAVR in connection with AF. Seeger et al. recently published a clinical follow-up study, reporting a promising anticoagulation regime using apixaban in patients with AF after TAVR [28]. This strategy suggests that an early safety endpoint in patients with AF receiving apixaban was significantly less frequent compared with patients receiving a VKA [28].

Nonetheless, some analyses have revealed contradictory results. The BRAVO-3 trial revealed similar early outcomes across groups regardless of anticoagulant strategy (AF and SR) and demonstrated that AF was not associated with significantly higher risk of adjusted 30-day outcomes [29]. A possible reason could be that 29.5% of AF patients were treated with DAPT without OAC, while only 16.5% of patients were treated without OAC in our study. Therefore, an increased number of left atrial occlusions could be responsible for this discrepancy.

In a recently published subgroup analysis from the SOURCE-XT registry [30], a significantly higher incidence of major vascular complications and life-threatening bleeding was reported among patients with pre-existing AF. However, the anticoagulation strategy was not investigated in this study. Maan et al. [31] found no statistically significant differences in major vascular complications between SR and AF patients in a retrospective study of 137 patients. Although bleeding after TAVR was not considered. Chopard et al. [32] did not show any significant difference in the occurrence of major vascular complications within 30 days after TAVR between patients with pre-existing AF and those with SR based on their analysis of data from the FRANCE-2 registries. However, there was a noticeably lower overall rate of major vascular complications. Furthermore, the rate of life-threatening bleeding was not explicitly stated in this study.

### Bleeding complications

Overall, 7.4% of the patients experienced life-threatening bleeding, 3.5% had major bleeding and 19.8% had minor bleeding during the first 30 post-procedural days.

In the large TAVR studies and registry data already published, the rates of life-threatening bleeding have differed among transfemoral (or mainly transfemoral) cohorts based on the VARC/VARC-2 criteria. The reported bleeding rates have ranged from 1.2% in the FRANCE-2 registry [23] to 13.6% in the CoreValve High Risk study [33], with rates of 6.7% in the PARTNER-2 study [15], 4.0% in the ADVANCE study [25] and 3.8% in the SOURCE-XT registry [24].

In the PARTNER study, hemorrhages were classified as major or minor using the modified VARC-2 criteria rather than classifying as major life-threatening bleeding. This resulted in major bleeding rates of 10.9% in cohort A and 16.8% in cohort B [3, 5]. Recent data from the GARY registry showed a decrease in the incidence of major bleeding (bleeding requiring transfusion of ≥ two RBC units) of 30.6% in 2011, 27.6% in 2012 and 23.0% in 2013 [19]. The SURTAVI study concluded an incidence of life-threatening or major bleeding of 12.2% [7] due to both technical advancements and increased user knowledge regarding TAVR. Accordingly, the year data were acquired in published studies must also be considered when interpreting the results and also applies to nearly all complication rates.

Concerning the distribution of bleeding complications between the two groups within the first 30 post-procedural days, patients with AF had life-threatening bleeding three times more frequently than patients with SR. A similar difference was evident in the rates and temporal development of major vascular complications. Although this difference remained constant throughout the follow-up period, further bleeding complications were unlikely in all successful TAVR procedures not complicated by bleeding or vascular complications during the first 30 post-procedural days. Concerning the occurrence of major or minor bleeding, no significant difference between the two groups was demonstrated either within 30 days after TAVR or during the complete follow-up period. Considering that 93.2% of SR patients were treated with DAPT, these results may indicate that the bleeding incidence of 3.7% per year already described in the literature for DAPT therapy may also be applicable to TAVR patients [34]. The propensity matched analysis from the ITER registry indicated aspirin alone does not increase the risk of prosthetic valve dysfunction and reduces the risk of peri-procedural complications and the risk of 30-day all-cause death compared to DAPT therapy after TAVI in patients with SR [35]. Supporting results were shown by Ichibori et al., who suggested that treatment with aspirin alone is an acceptable regimen for TAVR patients with SR [36]. Although the risk of bleeding is lowered by a single antiplatelet therapy, valve thrombosis and dysfunction are frequently observed. Indeed, observational studies found subclinical leaflet thrombosis rates up to 13% [37]. Subclinical leaflet thrombosis occurred frequently in bioprosthetic aortic valves, more commonly in TAVR than in surgical valves [38]. Anticoagulation therapy (both NOACs and VKA), but not DAPT, was effective in preventing or treating subclinical leaflet thrombosis. Subclinical leaflet thrombosis was associated with increased rates of TIAs and strokes [37].

These collective findings indicate that there remains a great need for further clarification. Ongoing large prospective, randomized, multicenter studies such as the GALILEO Trial [39] are needed to analyze the combination of rivaroxaban and aspirin compared to aspirin and clopidogrel.

To date, few studies have specifically investigated the influence of AF on the occurrence of bleeding after TAVR. Stortecky et al. [40] demonstrated a comparable incidence of life-threatening and major bleeding among patients with AF and SR in a collective study of 389 patients, 131 of whom (33.7%) had AF. Interestingly, in this study, only 39% of AF patients received combined VKA and APT post-procedural therapy (the remainder of patients received aspirin or clopidogrel therapy). Of the patients in the present study, 67.9% of those with AF were treated with OAC plus APT for 3 months, which may explain the different occurrence rate of life-threatening bleeding. Tarantini et al. [30] similarly reported significantly more life-threatening bleeding in a group of AF patients within the first year after TAVR. Although the incidence of major bleeding was similar between the two groups in their study, minor bleeding was significantly more frequently observed among the patients with AF. Chopard et al. [32] considered life-threatening bleeding only as part of a 30-day combined safety endpoint (total mortality, stroke, major vascular complications, acute renal failure, coronary artery obstruction, and flap dysfunction requiring re-intervention) in a comparison between patients with AF or SR. However, it should be noted that the overall anticoagulant therapy regimen was not recorded in Chopard et al. [32] or Tarantini et al. [30]. Vavuranakis et al. [41] also observed no significant difference in the occurrence of major bleeding between patients with AF and SR in their small analysis of 80 patients (20 patients with AF and 20 with SR after propensity scoring). However, this result is likely due to the small number of patients. Furthermore, in contrast to the present study, Vavuranakis et al. did not analyze life-threatening or minor bleeding [41]. While anticoagulation strategies suggest a reduction in antiplatelet agents with SR [35], the optimal regime for patients with AF after TAVR remains to be addressed. Ongoing studies with apixaban (Atlantis) and edoxaban, such as the ENVISAGE-TAVI AF study, have been initiated to shed light on this gray area.

Seeger et al. evaluated the impact of AF on outcomes in TAVR and evaluated the safety and efficacy of apixaban compared with VKA in patients with AF [28]. In agreement with our results, they showed a significantly higher rate of all-cause mortality throughout the 12-month follow-up in patients suffering from AF undergoing TAVR. Moreover, the study of Seeger et al. included more patients treated with the Xa inhibitor apixaban than our study (data not shown). While Seeger et al. found more life-threatening bleeding events but no difference in major vascular complications in patients with AF, the number of major vascular bleeding events was not separately shown. Seeger et al. evaluated after 4 weeks of treatment with a combination of APT and either NOAC or VKA the single use of an anticoagulation regime without platelet inhibitors. Single or double antiplatelet regimes were prescribed according to the valve type.

For the first time, pre- and post-procedural (within 48 h after TAVR) hemoglobin levels (Hb) were compared in this study for a more precise analysis and better understanding of the cause and extent of bleeding. A post-procedural Hb drop of 14.4 ± 11.7% was observed in the entire patient population. Interestingly, patients with SR had relatively greater post-procedural Hb level reductions than those with AF. This finding could be explained by the fact that the AF group received more RBC transfusions due to more life-threatening bleeding.

### Cerebrovascular events

Since the first TAVR, neurological complications have been a primary concern, with particular care dedicated to prevention. Notably, in the PARTNER study, the overall rate of neurological events after TAVR was twice that of conventional aortic valve repair [5]. Furthermore, cerebral magnetic resonance tomography revealed new, although usually asymptomatic, cerebral lesions in the majority of patients after TAVR [42, 43].

The stroke rates within 30 days and 1 year after TAVR in this study were 3.1 and 5.0%, respectively, comparable to reported rates of 1.5–4.2 and 4.1–6.9%, respectively, in previously published TAVR studies and registries [15, 19, 23–25, 44]. Furthermore, post-procedural transient ischemic attacks were observed in only 0.8% of our total patient population, consistent with the rates of below 1.0% reported in the literature [15, 25, 40]. The strategy of Seeger et al. using an Xa inhibitor instead of VKA was related to a significantly lower rate of early safety endpoint in patients with AF compared with patients treated with a VKA (13.5% vs. 30.5%; *p* < 0.01), with a lower stroke rate (2.1% vs. 5.3%; *p* = 0.17) at the 30-day and 12-month (1.2% vs. 2.0%; *p* = 0.73) follow-ups [28].

A limitation of the international data and of the present patient cohort is that only patients with symptomatic neurological deficits underwent MR or CT investigations. Accordingly, subclinical strokes may not have been detected.

Although AF is the most common cause of embolic stroke [16], no significant differences in the incidence of stroke were detected within 30 days or within 1 year after TAVR between the AF and SR study groups. Additionally, the number of patients who suffered a TIA did not differ significantly between the two groups. This result may be attributable to the good post-procedural anti-coagulant therapy for patients with AF. Fortunately, no elevated rates of cerebral bleeding were observed during the observation period.

### Mortality associated with TAVR

The overall 30-day and one-year mortality rates in this study were 4.7% and 21.0%, respectively, with most deaths attributable to cardiovascular events. Accordingly, the corresponding cumulative 30-day and one-year cardiovascular mortality rates after TAVR were 3.9% and 17.4%, respectively. Considering pre-existing cardiac rhythm, patients with AF in this study experienced significantly higher cardiovascular and overall mortality than patients with SR. These findings can be explained mainly by the more frequent occurrence of severe complications (life-threatening bleeding, major vascular complications) among patients with AF. The results of the present study and the subgroup analyses to date underscore the need to include AF in future risk scores, particularly for TAVR, and to optimize the post-procedural management of these patients, including anticoagulant therapy as appropriate, to avoid the complications of TAVR.

No prospective, randomized study has been conducted thus far to evaluate antithrombotic therapy after TAVR among patients with AF. Until now, guidelines have been derived from retrospective studies and meta-analyses [14, 18, 28, 45–47]. As a result, anticoagulation regimens associated with TAVR are primarily either empirical or are administered at the discretion of the attending physician [48, 49]. The observed diversity of treatment regimens confirms the clinical challenge of selecting post-procedural medications, even among the patients in this study. The two anticoagulation regimens for patients with AF in our study included VKA plus clopidogrel or NOAC plus clopidogrel for 3 months. These two treatment subgroups were studied for mortality and complications after TAVR. Subgroup analyses were not conducted for other treatment regimens due to the small group sizes in this study. Considering the analysis of this retrospective data, the selection criteria for treatment with either VKA or a NOAC must be questioned. According to the statistical analysis of the baseline characteristics, patients in the NOAC group were significantly older than patients in the VKA group. Additionally, they had a higher bleeding risk based on their HASBLED scores. This observation could reflect the fact that older patients with increased bleeding risk have commonly been treated with an NOAC due to the difficultly of regular INR monitoring necessary for therapeutic VKA anticoagulation. Regarding the complication and mortality rates after TAVR during the follow-up period, no significant differences between the two groups were found in this analysis. These results support the future use of NOAC after TAVR. However, it is also important to note that the patients in the NOAC and VKA groups underwent implantation during different time periods. Specifically, the earliest patients with AF (2010) were treated with VKA, whereas NOACs, although not recommended in standard guidelines, were preferred after 2014.

To recommend the findings of Seeger et al. [14] and the results from this study for patients with SR, we anticipate the results of future randomized, prospective, large studies, such as the GALILEO trial [16]. Furthermore, this topic of interest is currently being investigated in multicenter, phase IIIb, prospective, open-label, randomized trial studies (ClinicalTrials.gov Identifier: NCT02664649) using apixaban or lixiana (Edoxaban Compared to Standard Care After Heart Valve Replacement Using a Catheter in Patients With Atrial Fibrillation (ENVISAGE-TAVI AF); ClinicalTrials.gov Identifier: NCT02943785) to determine the advantages of the current standard of care for patients with AF undergoing a successful TAVR procedure.

We believe that the unidentified risk for valve thrombosis and subclinical stroke might be adequately addressed with oral anticoagulation agents. In the future, patients undergoing TAVR might benefit from post-intervention anticoagulation therapy in combination with an NOAC, and possibly at a reduced dose in patients with concomitant disease in other vascular beds according to the results of the COMPASS trial [17]. However, the optimal dose and duration of therapy still need to be clarified. Thus, it is recommended to adhere to current guidelines, with routine DAPT and recourse to OAC when specifically indicated, while tailoring therapy based on bleeding and thromboembolic risk in individual patients [50].

## Conclusion

The presence of AF was associated with markedly increased risk for life-threating bleeding and major vascular complications in patients with aortic valve stenosis treated with TAVR. AF had an independent detrimental effect on one-year overall mortality after TAVR. More data are needed to define the role of AF prevention and treatment on outcomes in these patients. Additionally, optimization of the anticoagulation strategy is warranted. Finally, the implementation of more comprehensive TAVR risk scores, taking into account AF, remains a relevant clinical need. Prospective studies may further clarify this phenomenon.

## Abbreviations

AF: Atrial fibrillation
APT: Anti-platelet therapy
CI: Confidence interval
DAPT: Dual anti-platelet therapy
HR: Hazard ratio
NOAF: New-onset atrial fibrillation
OAC: Oral anticoagulation
OR: Odds ratio
PAD: Peripheral arterial disease
PCI: Percutaneous coronary intervention.
RBC: Red blood cell
SR: Sinus rhythm
TAVR: Transcatheter aortic valve replacement
TIA: Transient ischemic attack
VKA: Vitamin K antagonist
